# Management of Cervical Fibroid during the Reproductive Period

**DOI:** 10.1155/2013/984030

**Published:** 2013-09-15

**Authors:** Remon Keriakos, Mark Maher

**Affiliations:** Department of Obstetrics and Gynaecology, Sheffield Teaching Hospitals, Royal Hallamshire Hospital, Jessop Wing, Sheffield S10 3QZ, UK

## Abstract

This is a case report of a 29-year-old lady who presented with excessive vaginal discharge and sessile cervical fibroid arising from the vaginal portion of the cervix. She was not suitable for uterine artery embolization as she has never previously been pregnant before. She was encouraged to get pregnant and to avoid surgical excision which can lead to hysterectomy. Shortly after, she became pregnant. She had many admissions during pregnancy due to bleeding from the fibroid, and in one occasion she had blood transfusions. The fibroid increased in size to become larger than the head of the baby. An emergency caesarean section was performed at 37 weeks when she attended in labour before the date of her elective caesarean section. She was managed conservatively following delivery in the hope that the fibroid becomes smaller making surgery easier. The fibroid degenerated and reduced in size. Vaginal myomectomy was carried out. The patient is now pregnant for the second time and had a cervical suture at 20 weeks gestation. In this educational case report we discuss the different management options of cervical fibroids and review the literature of other similar cases and their outcome.

## 1. Introduction

A cervical fibroid during pregnancy is rare. There are only very few reported cases in the literature. Sessile cervical fibroid, arising from the vaginal portion of the cervix, in pregnancy is extremely rare, and to our knowledge there are only three cases reported in the literature. 

Although the majority of fibroids (60%–78%) show no significant change in size during pregnancy [1], some may rapidly increase in volume due to the increased blood flow to the uterus and the high levels of steroid hormone [2]. Antenatal and postnatal complications can arise depending on the size and type of the fibroid. Surgical interventions during pregnancy have been described in certain types of cervical fibroids. 

In this case report we discuss management of sessile cervical fibroid arising from the vaginal portion of the cervix before pregnancy, during pregnancy, during delivery, and after delivery and management of patients' future pregnancy. 

## 2. Case Report

**Figure 1:**
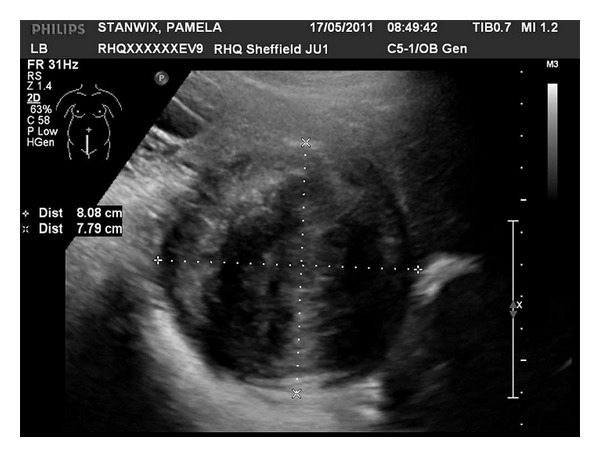
Cervical fibroid size at 20 weeks 8.08 × 7.79 cm.

**Figure 2:**
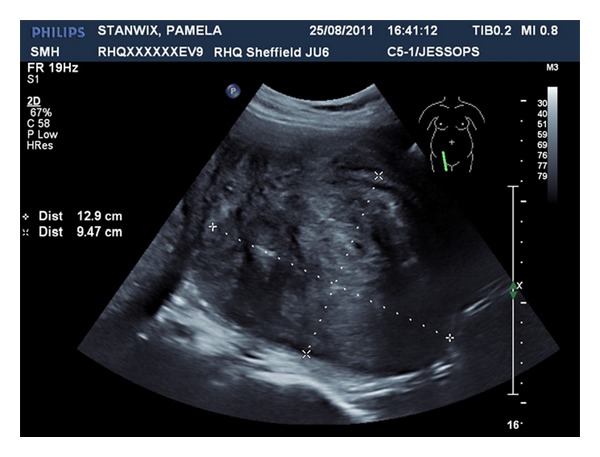
Cervical fibroid size at 36 weeks 12.9 × 9.47 cm.

A 29-year-old nulligravida lady was referred to gynaecology clinic complaining of significant vaginal discharge for 8 months sufficiently heavy to warrant frequent changing of her panty liner. During the same period, the patient had several episodes of intermenstrual light vaginal bleedings. She was on oral contraceptive pills for 3 years, and the bleeding and the vaginal discharge did not change with cessation of the pills. She was screened for sexually transmitted diseases by her general practitioner, and the results were normal. Two large loop excisions of the transformation zone for intraepithelial neoplasia had previously taken place. On clinical examination, she was found to have a 50 mm sessile cervical fibroid arising from the posterior wall of the left cervical lip of the cervix. This was confirmed by vaginal ultrasound scanning. The patient was put off uterine artery embolization due to the small risk of ovarian failure and risk of bleeding and infection which could lead to a hysterectomy. Hence, she was advised to consider starting to try and conceive sooner rather than later. Eight weeks later she got pregnant. She had a scan at 7 weeks which confirmed viable intrauterine pregnancy. She had regular antenatal care but experienced recurrent vaginal bleeding throughout the pregnancy due to her cervical fibroids and hence, she had several admissions to the antenatal ward. Her haemoglobin was regularly monitored, and she was placed on prophylactic iron therapy. On one occasion, at 20 weeks gestation, haemoglobin levels had reached 8.4 gm/dL, and 2 units of blood were transfused. The patient was treated conservatively, and a plan of elective caesarean section was agreed on at 39 weeks gestation, but she was aware that this might be brought earlier if she developed heavy bleeding before the agreed on date. At 20 weeks the size of the fibroid increased to 81 × 78 × 88 mm (Figure 1). Furthermore, at 36 weeks the size of the fibroid increased to 129 × 95 × 112 mm (Figure 2), which is larger than the size of the baby's head, distending and filling the entire vagina. The fetal growth was normal.

At 37 weeks gestation she went into spontaneous labour and had an emergency caesarean section. A live male infant weighing 6.5 lbs was delivered in good condition. The immediate postoperative period was uneventful, and the patient began breast feeding whilst in hospital. The patient was then sent home with an appointment for an ultrasound scan in 8 weeks with the hope that the fibroid will decrease in size. She was informed that the fibroid may degenerate due to spontaneous thrombosis of the feeding blood vessels. However, the patient attended the gynaecology emergency admissions at 6 weeks following delivery with offence vaginal discharge, passing small pieces of tissues. On examination, there was excessive offensive vaginal discharge and the size of the fibroid mass reduced to 50 mm, appearing necrotic in some areas. She was given clindamycin vaginal cream and an appointment to come for an outpatient clinic a week later for a biopsy of the tumour. Histology confirmed cervical myoma and areas of degenerations. She was admitted 4 days later and had a cervical myomectomy via vaginal route. The entire myoma was enucleated. 

She was reviewed in the gynaecology clinic 6 weeks later.

The cervix had healed well but appeared short. This might be due to the previous two cervical loop excisions for intraepithelial neoplasia of the cervix, in addition to cervical myomectomy. It was decided that cervical length monitoring will be needed during any further pregnancies from 14 weeks onward. 

At the time of writing this case report, we were made aware by the patient that she is currently in her second pregnancy at 20 weeks gestation. She had a cervical suture at a different hospital in another town as she moved her place of residence. The scan revealed that the cervix is shortening. She was also placed on progestogen vaginal tablets.

## 3. Discussions

Cervical fibroids can affect the supravaginal or vaginal portion of the cervix. There are several types of cervical fibroid and each can present differently. Supravaginal fibroids can be central surrounding the entire cervical canal and lying centrally in the pelvis displacing the uterus superiorly. They can also be unilateral or bilateral, can be intramural or subserosal, and can be lying in the pelvis. If women get pregnant with these types of fibroid, the mode of delivery would be by caesarean section especially with central cervical fibroids. The size of the fibroid might increase significantly during pregnancy displacing the lower segment high up, and in these cases the abdominal incision should be via midline incision. Patients may suffer from pressure symptoms and pain in pregnancy. These are most frequently seen during the second and third trimesters of pregnancy in women with large fibroids (>5 cm) [2]. Management during caesarean sections should be conservative. Future management of these fibroids is usually by hysterectomy especially for central cervical fibroids. Uterine artery embolization and myomectomy can be performed depending on patients' symptoms, fertility desire, the site of the mass, and associated uterine fibroids.

Pedunculated cervical fibroids can arise from the endocervical canal or from the uterine cavity and protrude through the cervix. These can be very large and cause recurrent vaginal bleeding during pregnancy with the option of being removed vaginally during the pregnancy [3].

Sessile cervical fibroids arising from the cervical lips of the vaginal portion during pregnancy are very rare with only 3 cases reported in the literature [4, 5]. They can rapidly increase in size [5] as in our case and lead to recurrent vaginal bleeding necessitating blood transfusion as in our case. There is also a risk of spontaneous rupture of the membranes (SROM) [4]. The management of the fibroids following delivery can be by myomectomy via abdominal route through an incision in the vagina during the caesarean section [5] or vaginal myomectomy as in our case. In our case we initially planned to manage the patient conservatively hoping that the fibroid degenerates by spontaneous thrombosis of its feeding blood vessels making myomectomy easier to perform. As we anticipated, this is exactly what had happened with the size of the fibroid reducing from 12 cm to 5 cm making it easier to remove with insignificant amount of bleeding. There is a risk of infection with degenerating fibroids, and these patients need monitoring for such risks in order to instigate early treatment as happened in our case. In two earlier similar case reports from 1958 [4], they were managed by abdominal hysterectomy; one had SROM at 20 weeks gestation with cord prolapse and intrauterine fetal death. She had abdominal hysterectomy following infection. The other case was delivered by caesarean section at 37 weeks gestation followed by caesarean hysterectomy. Uterine artery embolization is a possibility in these cases to help reducing the size of the fibroid before the myomectomy.

In future pregnancy, women should be scanned for cervical length and to insert cervical suture to reduce the risk of miscarriage as in our case. 

## 4. Conclusion

This case report has shown that women with sessile cervical fibroids can be managed conservatively before pregnancy, during pregnancy, and after delivery with very good outcomes. Myomectomy can be delayed following delivery as these fibroids decrease in size making it easier to remove them vaginally. Future pregnancies should be monitored with cervical length measurement.

## References

[1] Aharoni A, Reiter A, Golan D, Paltiely Y, Sharf M (1988). Patterns of growth of uterine leiomyomas during pregnancy. A prospective longitudinal study. *The British Journal of Obstetrics and Gynaecology*.

[2] Katz VL, Dotters DJ, Droegemueller W (1989). Complications of uterine leiomyomas in pregnancy. *Obstetrics and Gynecology*.

[3] Oruc S, Karaen O, Kurtul O (2004). Coexistence of a prolapsed pedunculated cervical myoma and pregnancy complications: a case study. *Journal of Reproductive Medicine*.

[4] Abitbol MM, Madison RL (1958). Cervical fibroids complicating pregnancy. *Obstetrics and Gynecology*.

[5] Erian J, El-Toukhy T, Chandakas S, Kazal O, Hill N (2004). Rapidly enlarging cervical fibroids during pregnancy: a case report. *Journal of Obstetrics and Gynaecology*.

